# A Scoping Review of Trauma-Informed Interventions and Violence Prevention in K-12 Schools in the United States

**DOI:** 10.1007/s12310-026-09874-2

**Published:** 2026-04-09

**Authors:** Briana A. Scott, Sarah M. Stilwell, Heather Murphy, Esther Lee, Melia Schliebe, Zaida V. Pearson

**Affiliations:** 1 Institute for Firearm Injury Prevention, University of Michigan, 1109 Geddes Ave., Ann Arbor, MI 48109 USA; 2 School of Public Health, University of Michigan, 1415 Washington Heights, Ann Arbor, MI 48109 USA; 3 Department of Educational Psychology, University of Illinois Chicago, 1040 W Harrison St, Chicago, IL 60607 USA; 4 Trinity Health, Ann Arbor, MI USA

**Keywords:** Trauma-informed, School violence, School programs, School mental health, Scoping review

## Abstract

More than two-thirds of children report experiencing at least one traumatic event by the age of 16. Childhood trauma can negatively affect youth’s physical and mental health, learning, relationships, and behavior, with implications for their long-term development. Schools are uniquely positioned and equipped to help youth cope with their trauma and prevent further traumatic events from occurring through programming, policy, and staff training. This scoping review investigates the relationship between trauma-informed school-based efforts and violence prevention in K-12 schools in the United States over the past twenty years (2004–2024). We conducted our review in accordance with PRISMA guidelines and limited our search to peer-reviewed articles published in the following databases: Education Abstracts, ERIC, PsycINFO, PubMed, and Scopus. To be included, studies must meet the criteria for a trauma-informed intervention based on the framework employed by Avery and colleagues (J Child Adolescent Trauma 14(3):381–397, 2021). Findings from the review suggest that trauma-informed school-based efforts (N = 7), such as social-emotional learning curricula, multi-tiered school-level models, and culturally relevant programs, are related to reduced violence in schools. Findings point to how school-based, trauma-informed interventions might be one avenue to addressing school violence and improving school safety, given the role of trauma as both a precursor to and consequence of violence.

**Supplementary Information:**

The online version contains supplementary material available at 10.1007/s12310-026-09874-2.

## Introduction

School violence is a significant public health concern in the United States (U.S.), with far-reaching consequences for students’ academic success, mental health, and safety (Espelage et al., 2013; Henry et al., 2010). Violent incidents in school, including bullying, physical fights, and weapon-related threats, contribute to an unsafe learning environment that affects students’ ability to thrive both socially and academically (Kutsyuruba et al., 2015). The urgency of addressing school violence has led researchers, educators, and policymakers to explore various intervention approaches, including social-emotional learning (SEL) programs, punitive disciplinary measures, and restorative justice approaches (Skiba et al., 2022). Among these, trauma-informed interventions have emerged as a promising approach to mitigating violent behaviors by addressing the underlying individual psychological and social factors contributing to aggression and conflict (Overstreet & Chafouleas, 2016). Further, universal trauma-informed interventions may support students who have been victims of violence or other forms of trauma in or outside of the school environment (Bethell et al., 2014).

Students who have been affected by trauma are more likely to struggle at school in numerous ways, including their ability to pay attention, retain and recall information, and socialize with peers (Denicola et al., 2025; Moore & Stoddard, 2025). Furthermore, existing school programming falls short of being implemented across ecological levels (Bronfenbrenner, 1994) and may not be attuned to the unique needs of students who have experienced trauma; this may lead students to feel isolated and develop a negative perception of their school community (Brunzell et al., 2019). By recognizing the profound impact of trauma on students’ behavior and fostering supportive, resilient environments, trauma-informed strategies seek to reduce school violence, improve emotional regulation, and enhance overall school climate (Brunzell et al., 2019; Thomas et al., 2019).

Trauma does not exist in a vacuum and may be viewed through the social ecological model, which asserts levels of intervention at the intrapersonal, interpersonal, institutional, community, and public policy (Bronfenbrenner, 1994; Pember & Pettit, 2025). Implementation across multiple levels of the socioecological model is important, as trauma and violence stem from interconnected factors at the individual, interpersonal (e.g., peer and family relationships), institutional (e.g., school policy and climate), community, and structural policy levels (Pember & Pettit, 2025). Much of the current research on trauma-informed interventions focuses on the individual and interpersonal socioecological levels (Maynard et al., 2019; Thomas et al., 2019); less is known about interventions at other ecological levels, including the institutional (e.g., school) level. Thus, the purpose of the present study is to conduct a scoping review of the literature to identify patterns, gaps, and implications for future research regarding trauma-informed interventions and school violence prevention in the U.S.

### Childhood Trauma

According to the Substance Abuse and Mental Health Services Administration (SAMHSA), trauma encompasses an event, series of events, or set of circumstances experienced as physically or emotionally harmful or life-threatening with lasting adverse effects on the individual’s functioning and mental, physical, social, emotional, or spiritual well-being (SAMHSA, 2014). More than two-thirds of children report experiencing at least one traumatic event by the age of 16 (Copeland et al., 2018). These experiences of trauma can occur at multiple ecological levels, including the individual level (such as physiological or physical abuse and neglect); the relationship level (such as sexual abuse and witnessing or experiencing intimate partner violence); the community level (such as school or community violence and exploitation); and the societal level (such as national disasters or terrorism, refugee or war experiences, and military family-related stressors, as well as the sudden or violent loss of a loved one; Copeland et al., 2007).

Adverse effects from the traumatic event may occur immediately or experience delayed onset, with symptoms that may include the inability to cope, hypervigilance, an increased state of arousal, changes in cognitive processes, and more, depending on the individual’s unique symptoms and experiences (SAMHSA, 2014). Adverse childhood experiences (ACEs), including exposure to abuse, neglect, domestic violence, and community violence, have been linked to emotional dysregulation, increased aggression, and difficulties with interpersonal relationships in school-aged children (Anda et al., 2006; Felitti et al., 1998). Researchers have demonstrated that students with high ACE scores are at a greater risk of engaging in disruptive behaviors, experiencing school disciplinary actions, and being involved in violent incidents (Bethell et al., 2014). However, it is important to note the limitations of research on ACEs, which include a history of relying on retrospective self-reports of adults and are consequently subject to recall bias (Kelly-Irving & Delpierre, 2019). While there is limited research regarding youth reports of ACEs and school violence, research by Forster and colleagues (2017) demonstrates a strong relationship between ACEs and the probability of school-based victimization and perpetration.

While trauma often leads to negative consequences for those who experience it, not all survivors of trauma have adverse outcomes or need intervention, and the signs and symptoms of trauma can manifest differently in children, adolescents, and adults. For example, children may experience difficulty concentrating, sleeping, and eating, as well as fear separation from caregivers (Fekutti et al., 1998; Finkelhor et al., 2015). At the same time, adolescents may also start engaging in risky behaviors, use substances, and experience changes in mood, such as feelings of depression (Flannery et al., 2004). The lasting effects of trauma can be seen through difficulties with learning, lower grades, higher rates of exclusionary discipline in school, increased involvement with the juvenile justice system, and long-term health problems (SAMHSA, 2023). The effect of ACEs can be partly determined by an individual’s emotional experiences and the availability and allocation of social support and school resources (SAMHSA, 2014). Traditional school disciplinary approaches, such as exclusionary punishments like suspensions and expulsions, and zero-tolerance policies, often fail to address the root causes of these behaviors and may exacerbate the cycle of trauma by alienating vulnerable students from critical support systems (Skiba et al., 2014). Alternatively, trauma-informed interventions seek to create school environments that acknowledge the prevalence of trauma, foster resilience, and implement strategies to de-escalate conflicts while promoting positive behavioral outcomes.

### School-Based Violence & Interventions

Trauma is ubiquitous among K-12 students, affecting their emotional and educational development, and tragically, exposure to potential trauma is not limited to experiences outside of schools (Duane et al., 2023). K-12 school-based violence encompasses a spectrum of aggressive behaviors, ranging from verbal harassment to physical assault, occurring within educational settings (Turanovic & Siennick, 2022; Turanovic et al., 2022). This form of violence can manifest in various forms, including bullying, harassment, physical altercations, and even more severe incidents such as school shootings (Turanovic & Siennick, 2022; Turanovic et al., 2022). School-based violence encompasses actions perpetrated by students against their peers, as well as violence directed towards educators or other school staff members. According to the socioecological model, school violence cannot be understood in isolation but is shaped by multiple, interacting levels of influence, including the individual, relationships with family and peers, community contexts, and broader societal factors (Bronfenbrenner, 1994). For example, exposure to family or community violence (relationship and community levels) increases a student's risk of both experiencing and engaging in violence at school (Espelage et al., 2014; Finkelhor et al., 2015). Violence occurrences extend beyond the physical school building, with increasing rates of cyberbullying being reported, which is detrimental to students’ mental health, academic performance, and overall sense of safety and well-being within and outside the school environment (Cantone et al., 2015). The prevalence and impact of K-12 school-based violence make it a critical issue demanding immediate attention.

There are many deleterious implications of school-based violence that can cause trauma for students. School-based violence significantly impacts the physical and psychological well-being of students involved. Victims of bullying or assault may experience lasting trauma, leading to decreased academic performance, anxiety, depression, and even suicidal ideation (Cohen, 2021; Wang et al., 2020). Moreover, witnessing violence can instill fear and anxiety among students, creating an environment that is not conducive to learning (Herrenkohl et al., 2019). Furthermore, the effects of school-based violence extend beyond the immediate impact on individual students and schools. Researchers have found that individuals who experience or witness violence during their formative years are at higher risk of engaging in delinquent behaviors later in life (Thomas et al., 2019).

Additionally, the normalization of violence within school settings can perpetuate a cycle of aggression and conflict, contributing to broader societal issues such as crime rates and social instability (Benbenishkty et al., 2016). Students who belong to marginalized groups are especially vulnerable to school violence, partly due to the “in-group” and “out-group” dynamics that often manifest in school settings (Thomas et al., 2019). For example, youth who identify as LGBTQ + are at an increased risk of being victimized in school (Myers et al., 2020). Further, it is essential to recognize that schools in the United States are established and operated within Eurocentric, colonial frameworks that may serve as sources of trauma for racial minority children, youth, and families. Without attentiveness to structural marginalization, including systemic racism, exclusion, and the reinforcement of white norms, trauma-informed practices risk perpetuating the very harms they seek to address. As Saleem and colleagues (2021) highlight, traditional trauma-informed approaches that center individual experiences without acknowledging the broader context of racial stress and trauma can inadvertently reinforce existing inequities and fail to foster genuine healing for marginalized students. Saleem and colleagues (2021) identify the need for trauma-informed frameworks that actively disrupt colonial and Eurocentric foundations, uplift resistance, and promote culturally responsive strategies that support the collective healing and empowerment of marginalized groups. Thus, considering an approach to trauma-informed schools that accounts for diverse student identities is imperative for ensuring the safety of all students.

Trauma-informed interventions at the institutional level in schools can play a critical role in mitigating the effects of trauma and reducing school violence, and such efforts to address mental health are often implemented through varying multi-tiered frameworks (Berger, 2019). One such framework, multi-tiered systems of support (MTSS), is a prominent framework for allocating in-school mental health services (Weist et al., 2014). In the MTSS framework, tier 1 supports are utilized universally for all students, tier 2 supports are intended for a subset of students (approximately 10–15% of students, for selective, targeted resources that need more intense support), while tier 3 is designed for approximately 5% of students who require individualized supports for students displaying signs of mental health concerns. Utilizing an MTSS framework for trauma-informed approaches provides the ability to integrate supports in varying levels of need within a school-based service delivery model (Chafouleas et al., 2016).

Numerous multi-tiered interventions have been implemented to address school-based violence. For instance, programs such as Positive Behavioral Interventions and Supports (PBIS) seek to prevent violence in schools by fostering positive behavior and creating safer environments, thereby improving social, emotional, and academic outcomes for all students (Horner et al., 2010). Other interventions, such as *Safe Dates*, *TakeCARE*, and *Second Steps*, focus on social and emotional learning to prevent relationship and gender-based violence in schools. These programs also educate students about bystander behavior and enhance their self-efficacy to intervene (Espelage et al., 2015; Foshee et al., 1998; Sargent et al., 2017).

Furthermore, various trauma-informed approaches in schools address trauma symptoms, mental health, academic performance, behavior, and social and emotional functioning. One example includes the HEARTS program, implemented in K-8th-grade schools within the San Francisco Unified School District, which supports academic success for trauma-affected students through a comprehensive three-tier intervention framework (Dorado et al., 2016). This whole-school approach includes modifying school policies, providing staff training, and addressing staff burnout and trauma with evidence-informed interventions (Dorado et al., 2016). Despite these efforts, no studies have yet examined the effects of trauma-informed interventions specifically on school-based violence outcomes.

### Trauma-Informed Practices in Schools

According to SAMHSA, a trauma-informed program, organization, or system realizes the widespread impact of trauma, recognizes signs and systems of trauma, responds by integrating knowledge about trauma into policy, procedures, and practices, and seeks to prevent re-traumitization (SAMHSA, 2014). Within this framework are six key principles of a trauma-informed approach: safety, trustworthiness and transparency, peer support, collaboration and mutuality, empowerment, voice and choice, and cultural, historical, and gender issues (SAMHSA, 2014). These principles guide the foundation for creating an approach focused on resilience. These principles can be demonstrated in school-wide initiatives through a safe and welcoming school climate and classroom, structured and predictable learning environments, and positive and attuned relationships (National Child Traumatic Stress Network, 2017).

Given the breadth and depth of trauma that students may experience at the intrapersonal, interpersonal, or community levels, schools have a vested interest in supporting the whole child (National Child Traumatic Stress Network, 2017). When schools approach students through a trauma-informed lens, educators are better equipped to provide educational and social-emotional support to bolster student success (Phifer & Hull, 2016). Trauma-informed practices have the potential to influence emotional, behavioral, and social responses (Phifer & Hull, 2016), creating an opportunity for school systems to provide equitable access to care for students. Trauma-informed approaches are grounded in principles of safety, trust, collaboration, empowerment, and cultural responsiveness (SAMHSA, 2014). Recent literature further clarifies how trauma-informed principles translate into practical, school-wide efforts that address not only student needs, but also those of staff and the school system itself. Avery and colleagues (2021) conducted a systematic review of school-wide trauma-informed approaches and identified three domains central to successful implementation: comprehensive staff professional development, school-wide practice change, and organizational restructuring of policy and procedures, defined below.

Staff professional development is important for equipping educators to recognize, understand, and respond to the effects of childhood trauma. Consistent, evidence-based training enables educators and other school staff to recognize signs of trauma, respond effectively, and create supportive learning environments. The Trauma Learning Policy Initiative’s (TLPI) Flexible Framework, posited by Cole and colleagues (2013), reinforces this by advocating for ongoing professional development, reflective supervision, and integration into professional learning communities, moving beyond one-time workshops to meaningful, sustained engagement. Despite these recommendations, Avery and colleagues (2021) note the literature frequently lacks detail regarding the depth and breadth of such professional development efforts, especially their long-term effects on outcomes such as school violence.

Practice change across the school, such as systematic screening for trauma exposure, paired with appropriate interventions and referrals, is another trauma-informed practice. Practice change may include integrating SEL curricula and restorative approaches, which can help buffer the impact of trauma and promote emotional safety, supportive relationships, and emotion regulation. The TLPI Framework emphasizes that classroom strategies should be adapted for trauma sensitivity, such as predictable routines, flexible responses, and reduced exclusionary discipline, which not only supports affected students but creates universal benefit across the entire student body (Cole et al., 2013). However, a gap in the literature remains regarding how trauma-informed practice changes can affect school violence outcomes. Finally, organizational change in policy and procedures can help schools, as institutions, become more trauma-informed. Avery and colleagues (2021) found that schools may revise disciplinary policies, embed trauma-informed language in mission statements, and establish school-wide protocols for responding to behavioral crises. By integrating these principles into classroom management and school policies, educators have the potential to create environments that reduce the likelihood of violence and promote prosocial behaviors among students.

### Current Study

Despite the growing interest in trauma-informed interventions, there remains a lack of consensus on the relationship with school violence outcomes (Maynard et al., 2019; Thomas et al., 2019). Researchers have examined the effects of specific programs, including trauma-sensitive schools and cognitive-behavioral interventions; however, findings vary in methodological rigor, sample size, and contextual factors (Berger et al., 2019; Jaycox et al., 2019). Furthermore, research has often focused on individual-level behavioral or academic outcomes rather than school-wide changes in rates of violence. Given the complexity of school violence and the multifaceted nature of trauma-informed approaches, we conducted a scoping review of the literature to synthesize the available evidence, identify trends, and highlight areas for future research. Specifically, the present scoping review seeks to (1) identify trauma-informed interventions implemented in K–12 school settings, map each intervention to its underlying trauma-informed principles, and synthesize the available evidence on how these interventions are associated with violent incidents in schools, and (2) highlight gaps in the literature to warrant future exploration. By providing a comprehensive overview of current research, this scoping review will contribute to the ongoing discourse on school safety and inform evidence-based policymaking and practice.

## Method

### Search Strategy

For this review, we conducted a scoping review in accordance with Joanna Briggs Institute (JBI) methodology guidelines and reviewed the empirical literature focused on trauma-informed interventions in K-12 schools (Moher et al., 2009; Tricco et al., 2018). We selected a scoping review methodology to comprehensively map the breadth and diversity of trauma-informed interventions and violence prevention approaches in K–12 schools in the U.S, rather than to evaluate intervention effectiveness or synthesize outcomes, as would be typical of a systematic review. This approach aligns with our inclusion of U.S.-based studies and timeframe restrictions, allowing us to highlight current trends, identify gaps, and inform future research and practice in this rapidly evolving field. We identified studies using a protocol informed by the Preferred Reporting Items for Systematic Reviews and Meta-Analysis (PRISMA), and searched research databases, screened published studies, applied inclusion and exclusion criteria, and selected relevant literature for the review; however, the research protocol was not registered in a formal database (Moher et al., 2009). With the support of a trained librarian, the comprehensive electronic search of publications used the following databases: Education Abstracts, ERIC, PsycINFO, PubMed, and Scopus. We restricted the search to English-language studies and limited database results to the past 20 years (2004–2024) to focus on the modern era of trauma and trauma-informed school-based research, which has developed rapidly over this period. The search terms addressed the main concepts of the search strategy: trauma AND K-12 school AND intervention AND school violence (see https://osf.io/yjkqz/ for search terms).

### Inclusion Criteria

To be included in this review, empirical studies had to report on a trauma-informed intervention within K-12 schools. We define trauma-informed interventions as having one or more of the following principles: (1) staff professional development directly related to understanding trauma and its effects, (2) practice change across the school, including changes in school behavior practices (e.g., trauma screening or intervention geared toward addressing trauma), or (3) organizational change in policy or procedures to create a trauma-informed school environment (Avery et al., 2021). We did not have inclusion criteria regarding tiers of interventions; thus, interventions that fall within tier 1, 2, or 3, as well as those that were not identified within a MTSS framework by the authors, were eligible for inclusion. These interventions must also focus on reducing school-based violence, only including violent behaviors that occur within school settings (e.g., intimate partner violence and neighborhood gang violence were not included in this review). Furthermore, articles must be peer-reviewed, conducted in the United States, and published in English. Following these inclusion criteria, we excluded articles that: (1) Did not report on an intervention; (2) Intervention was not trauma-informed; (3) Did not occur within a K-12 school setting; (4) Did not report on a school violence outcome that took place within the school; (5) Were conducted outside of the United States; (6) Were not published in English; (7) Were a review, editorial, opinion piece, commentary, book, or book review.

### Study Selection

We used Covidence, an online platform, to manage the screening and selection of articles. The primary coding team was composed of three members: two senior researchers with expertise in school violence prevention, school-based program evaluation, and systematic review methodology (Coders A and B) and one master’s-level researcher with professional experience in addressing trauma in schools (Coder C), who received training from the senior team. Coders A and B collaborated with a librarian to develop and implement the search strategy. Coder C joined the process at the screening stage, receiving structured instruction from Coders A and B. Research assistants also assisted with article screening and were trained by the senior team. All titles and abstracts were independently and blindly screened by two coders to determine eligibility. Discrepancies in screening decisions were discussed and resolved by consensus during team meetings. The same team members conducted the full-text screening to determine article inclusion in the final sample. After completing the full-text review, the team established standardized criteria and protocols for data extraction from eligible studies. Initial data extraction was performed by two team members and subsequently reviewed by a third member for quality and accuracy. Throughout each phase, the research team convened regularly to address and resolve any disagreements, ensuring that all inclusion and exclusion decisions were made unanimously.

### Data Extraction

We developed a data extraction procedure for information from the articles that passed full-text review and met the inclusion criteria. We extracted the study author and publication year, the geographical location of the study (to the degree of specificity reported by the authors), the school level (i.e., elementary, middle, high school); intervention audience (i.e., students and/or parents); study design (i.e., randomized control trial); intervention description; and trauma-informed principles of the intervention (staff professional development, practice change, and/or organizational change). Finally, we extracted the school violence outcome, as described by the author, that the intervention sought to address, and briefly reported the results of each intervention.

## Results

**Fig. 1:**
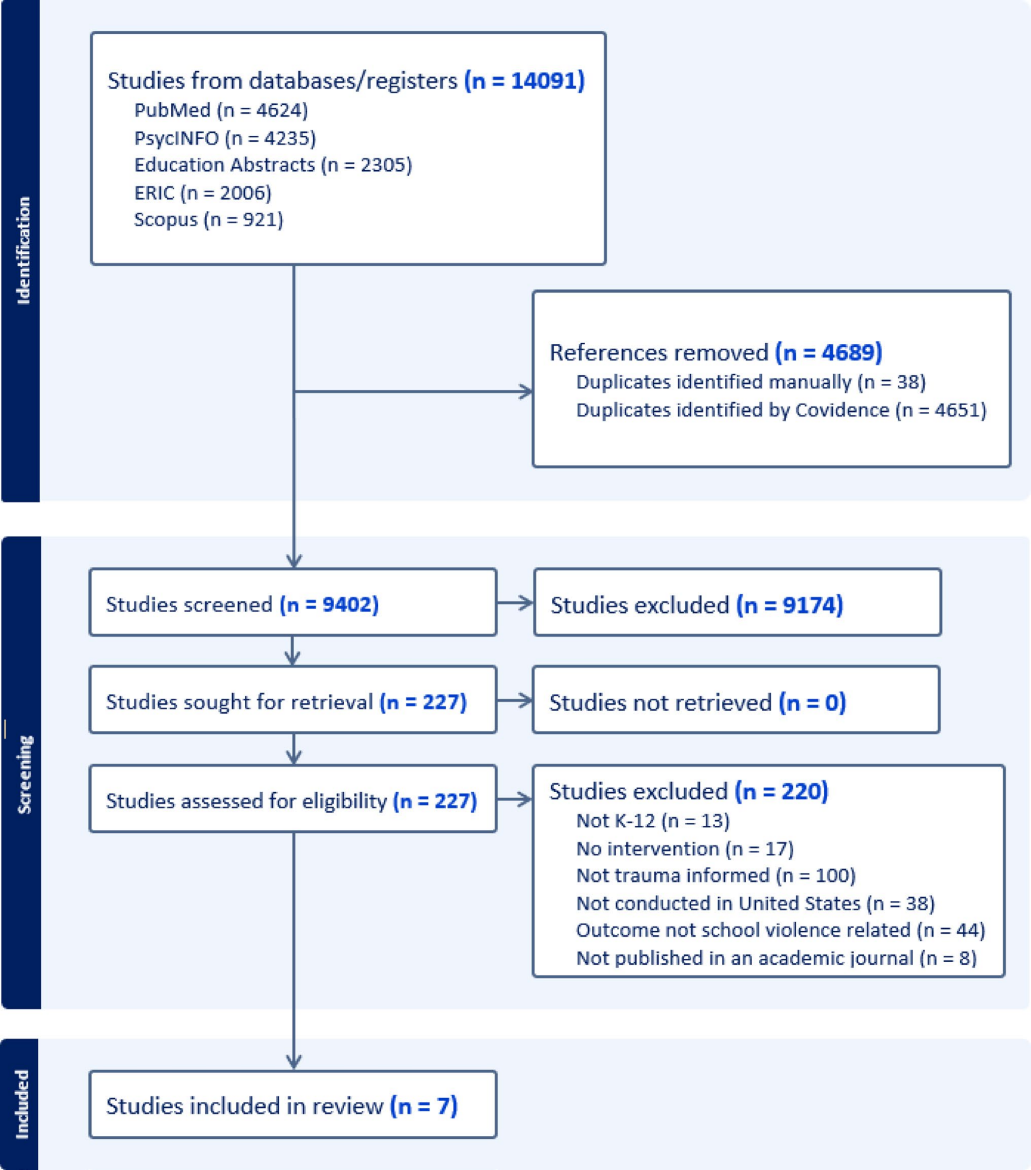
PRISMA flow chart

Figure 1 depicts our systematic review process. The initial database search identified 14,091 articles, of which 4,689 were identified as duplicate records and removed. Next, 9,402 records were examined during the initial title/abstract screening, of which 9,174 were excluded due to not meeting the identified inclusion/exclusion criteria. A total of 227 records were examined for full-text review. Of these records, 220 were excluded due to not meeting the established inclusion/exclusion criteria. In total, the final sample consisted of 7 studies that were included in the review. Characteristics of these studies are represented in Table 1.

**Table 1:** Description of articles (N = 7)

Author, year	Study setting & school level	Audience	Study design	Description of intervention	Trauma-informed principles	School violence outcome	Results
Domino, 2013	7th-grade students in CT	Students (N = 323) and parents	Pre-post test with control group	*Take the Lead (TTL)* is a 16-lesson SEL curriculum with optional workshops for parents	Practice change, Professional development	Bullying, Victimization	Participants in TLL reported reduced bullying and victimization compared to the control group
Dunn et al., 2020	Middle school boys in MA	Students (N = 8)	Pre-post test, focus groups	The *Healthy Power Program* is an 8-week violence prevention program for middle school-aged boys	Practice change, Organizational change	Conflict resolution, Physical violence	The focus group results suggest participants better understood conflict resolution; there was no change in quantitative data
Griffin et al., 2007	Middle school students in GA	Students (N = 336)	Randomized control trial	A universal, whole-school cognitive behavioral and cultural enrichment training	Practice change, organizational change, professional development	Motives, victimization, perpetration	The intervention was most effective when implemented with individualized training, cultural enrichment, and positive reinforcement
Kelly et al., 2010	Elementary school students grades 3–5 in South TX	Students (N = 312)	Randomized control trial	The *El Joven Noble* program prevents violence by developing a gender-equitable and nonviolent cultural identity	Practice change, organizational change	Attitudes about gangs, nonviolent self-efficacy, program values	Students with high-risk behaviors in the intervention group had greater self-efficacy and endorsement of program values than did high-risk students in the control group
Midgett & Doumas, 2020	6th-grade students in Northwestern US	Students (N = 147)	Pre-post test with follow-up	*STAC*, representing four strategies taught to students to intervene on bullying, is a 90-min bystander preventative intervention training followed by two 15-min booster sessions for middle school students	Practice change	Bullying perpetration	Participants in STAC reported a significant reduction in bullying perpetration from baseline to 6-week follow-up
Morgan-Lopez et al., 2020	6–8 grade, district in Southeastern US	Students (N = Not listed)	Randomized controlled Trial, Quasi-experimental	School-Based Mental Health (SBMH) programs (3 models) provide different levels of intervention services compared to non-SBMH schools; the Enhanced Model provides SPARCS and DBT treatment	Practice change	Aggressive behavior, Victimization	SBMH schools that expanded services demonstrated decreases in aggression and victimization
Swaim et al., 2008	Six middle schools (KY, LA, IL, ID, CA)	Students (N = 1,492; 780 controls, 712 interventions)	Randomized control trial	*Resolve It, Solve It* community and school-based anti-violence media campaign (media, peer role models, public education) delivered by older peers to address (1) respect for individual difference, (2) conflict resolution, and (3) anti-bullying	Practice change	Violent intentions, verbal assault, physical assaults against objects, physical assaults against people, verbal victimization, physical victimization	Significant differences between intervention and control students in growth rate for intent for violence, physical assault against people, verbal victimization, and perceived safety at school, but no differences for verbal assault, physical assault against objects, physical victimization, or self-efficacy (for avoiding violence)

SPARCS = Structured psychotherapy for adolescents responding to chronic stress, DBT =  Dialectical behavioral therapy

### Intervention Types and School Settings

Most interventions included in this review were implemented in middle schools, with a few at the elementary school level. No articles described interventions being implemented in high school settings. Interventions included school-based mental health programs (Morgan-Lopez et al., 2020), SEL curricula (Domino, 2013), bystander intervention training (Midgett & Doumas, 2020), and peer-led anti-violence campaigns (Swaim et al., 2008). Interventions ranged from brief sessions to comprehensive multi-week models that incorporated elements such as therapeutic techniques, conflict resolution, and cultural enrichment. Only one article (Morgan-Lopez et al., 2020) explicitly identified where programs were aligned within a MTSS framework; as such, a tiered framework was not an area used to compare articles within Table 1.

Structured SEL curricula were commonly implemented in middle schools, often consisting of multiple lessons designed to improve students’ emotional regulation and interpersonal skills. For example, the *Take the Lead* program (Domino, 2013) consists of a comprehensive curriculum for students as well as optional workshops for parents, promoting a collaborative, community-wide approach to fostering positive behaviors. Similarly, programs like *El Joven Noble* support elementary-aged students by developing gender-equitable and nonviolent cultural identities, offering a flexible yet structured approach to violence prevention (Kelly et al., 2010).

School-based mental health programs varied in intensity, with some models incorporating tiered levels of support (Morgan-Lopez et al., 2020). The programs included clinical services such as cognitive behavioral therapy, dialectical behavior therapy, and structured trauma interventions (e.g., Structured Psychotherapy for Adolescents Responding to Chronic Stress; SPARCS). These programs sought to support students affected by trauma and were often implemented across multiple grade levels.

### Trauma-Informed Principles

Interventions identified in this review incorporated trauma-informed principles through targeted practice changes, professional development for educators, and broader organizational reform, distinguishing them from generic approaches to school safety or behavior management. Practice change was central across several studies, with programs such as Take the Lead (Domino, 2013) implementing a 16-lesson SEL curriculum for seventh graders. Although not labeled explicitly as “trauma-informed” by the authors, this study met our criteria because the program goals align with core trauma-informed principles, such as strengthening emotional safety and supportive relationships and building students’ capacity to recognize and manage strong emotions, alongside lessons to explicitly address exclusion, aggression, and bullying. Consistent with a trauma-informed emphasis on reducing interpersonal threat and promoting relational safety in everyday school interactions, the program was designed to foster emotional safety and positive student–teacher relationships, resulting in reduced bullying and victimization.

Similarly, Midgett and Doumas (2020) reported on a bystander intervention, empowering middle school students with actionable strategies for intervening in bullying. This training led to a significant decrease in bullying perpetration, illustrating the trauma-informed principle of student empowerment and peer support. The School-Based Mental Health (SBMH) programs evaluated by Morgan-Lopez et al. (2020) provided trauma-focused therapies and expanded intervention services, leading to decreases in aggression and victimization.

Professional development for educators was another trauma-informed principle, evident in the study by Griffin and colleagues (2007), where individualized training and cultural enrichment deepened staff knowledge about trauma, enhanced relational trust, and shifted attitudes regarding student motives and behavior. This contrasts with generic professional development that centers exclusively on classroom management or discipline. Organizational change was also prominent, as seen with Dunn and colleagues (2020), whose *Healthy Power Program* combined practice change with systemic reforms to create safer environments and clear conflict resolution protocols, supporting both attitudinal and behavioral transformation. Additionally, Kelly and colleagues’ (2010) *El Joven Noble* program sought to develop gender-equitable and nonviolent identities among elementary students by embedding trauma-informed values in school policy and cultural norms, which increased self-efficacy and positive attitudes, especially among high-risk youth.

Organizational change in school procedures, as seen with the community initiative described by Swaim and colleagues (2008), further reinforced trauma-informed principles through media campaigns and older peer role models to promote respect for individual differences and conflict resolution. Distinct from interventions focused solely on reducing disruptive behaviors, these trauma-informed programs prioritized safety, empowerment, and attunement, affecting not only observable student outcomes like reduced aggression and bullying but also deeper attitudinal shifts that foster positive school-wide climate and relational trust. Together, these studies demonstrate the unique, multi-level integration of trauma-informed principles in school interventions, encompassing changes in attitudes, practices, staff capacity, and organizational structures to address both the causes and consequences of trauma in K-12 settings.

### Implications for School Violence

Interventions included in this review sought to address a variety of violence-related behaviors occurring at school, including: violence victimization (Domino, 2013; Morgan-Lopez et al., 2020), bullying (Domino, 2013; Midgett & Doumas, 2020), verbal and physical assault (Swaim et al., 2018), and aggressive behavior (Morgan-Lopez et al., 2020). A comprehensive list of all violence outcomes is represented in Table 1.

Findings suggest that trauma-informed interventions positively impact school violence outcomes, although results varied based on study design and implementation. Several studies reported reductions in victimization (Morgan-Lopez et al., 2020) and bullying (Domino, 2013; Midgett & Doumas, 2020), while others noted improvements in students’ conflict resolution skills (Dunn et al., 2020) and self-efficacy in managing aggression (Kelly et al., 2010). Whole-school cognitive behavioral interventions incorporating cultural enrichment and individualized training demonstrated greater success in reducing violence-related motives and behaviors (Griffin et al., 2007). Additionally, school-based mental health programs, such as those by Morgan-Lopez and colleagues (2020), demonstrated significant reductions in aggressive behaviors and victimization. Peer-led interventions, such as the *Resolve It, Solve It* campaign, decreased verbal victimization and influenced students’ perceptions of safety, although effects on physical victimization and assault were less consistent (Swaim et al., 2008). Moreover, results of interventions designed for high-risk behaviors, such as *El Joven Noble,* demonstrated marked improvements in self-efficacy and attitudes toward violence prevention (Kelly et al., 2010).

## Discussion

Based on the findings from the current scoping review, trauma-informed interventions in K-12 schools demonstrate promising effects on violence-related outcomes, including reductions in victimization, bullying, and aggression (Domino, 2013; Midgett & Doumas, 2020; Morgan-Lopez et al., 2020; Swaim et al., 2008). However, the evidence base remains limited, with most studies focusing on behavioral outcomes rather than directly examining students’ trauma exposure or trauma-specific symptoms (Avery et al., 2021). This gap underscores the complexity and individualized nature of trauma, suggesting that universal, one-size-fits-all approaches may not fully address the nuanced needs of diverse student populations (Phifer & Hull, 2016).

Schools are uniquely positioned to implement MTSS that integrates trauma-informed principles across universal (Tier 1), targeted (Tier 2), and intensive (Tier 3) levels. Universal interventions, such as SEL curricula (Domino, 2013), peer-led anti-violence campaigns (Swaim et al., 2008), and programs promoting cultural enrichment and self-efficacy (Kelly et al., 2010), provide foundational skills in emotional regulation, conflict resolution, and relationship building. These approaches align with the SAMHSA (2014) principles by promoting safety, trust, empowerment, and collaboration school-wide, and are effective in improving climate and reducing disruptive behaviors (Cole et al., 2013; National Child Traumatic Stress Network, 2017).

For future implementation within an MTSS framework, several components may be prioritized. First, structured professional development is critical. Interventions such as those described by Griffin and colleagues (2007) demonstrate that individualized teacher training and cultural enrichment foster education, relational trust, and support for both attitudinal and behavioral change. Comprehensive professional development helps educators recognize trauma’s effects, adopt trauma-sensitive responses, and sustain trauma-informed practices over time (Chafouleas et al., 2016; Hydon et al., 2015).

Second, practice change could be embedded at every tier. Evidence from both SBMH programs (Morgan-Lopez et al., 2020) and SEL-type curricula (Griffin et al., 2007) shows that integrating trauma-focused therapies and mental health services at the Tiers 2 and 3 supports students with greater needs. These programs, ranging from brief interventions to comprehensive treatment, are most impactful when combined with universal classroom strategies that build emotional safety and prosocial skills (Brunzell et al., 2019; Overstreet & Chafouleas, 2016).

Third, organizational change, including revisions to discipline procedures, creation of safe spaces, and the inclusion of trauma-informed language in school policies, has been shown to reinforce trauma-sensitive values and foster a positive school climate (Avery et al., 2021; Dunn et al., 2020; Kelly et al., 2010). Schools may also consider contextual variables such as resource availability, demographics, and local policy when designing or scaling up trauma-informed interventions (Stilwell et al., 2024).

Importantly, trauma-informed practices support not only students with high levels of trauma exposure but also the broader school community. By fostering supportive relationships, improving conflict resolution, and promoting self-efficacy, schools can reduce the potential for violence and contribute to healthier, more compassionate learning environments (Brunzell et al., 2019; Phifer & Hull, 2016). Teacher well-being is also enhanced through a trauma-informed approach, as regular self-care and collaborative professional learning communities prevent burnout and strengthen resilience among staff (Hydon et al., 2015).

Despite these advances, significant gaps remain. The field lacks large-scale, longitudinal studies that assess the implementation fidelity and long-term sustainability of trauma-informed approaches within MTSS frameworks (Avery et al., 2021; Cole et al., 2013). Future research might address how best to tailor interventions to varied student populations, clarify which components are most effective at each tier of MTSS, and examine mechanisms by which trauma-informed practices reduce school violence across ecological levels (SAMHSA, 2014; National Child Traumatic Stress Network, 2017). Integrating trauma-informed interventions within MTSS can enhance safety, promote resilience, and reduce violence in school settings. The reviewed studies provide limited but suggestive evidence that more comprehensive, multi-level approaches, linking professional development with shifts in practice and, in some cases, policy, may better align with trauma-informed principles than single-component efforts. Notably, only one of the seven studies incorporated professional development, practice change, and policy change concurrently; therefore, conclusions about the necessity of all three components should be interpreted cautiously.

### Limitations

Several limitations should be noted in the present study. First, this review did not include grey literature, such as reports, dissertations, or unpublished studies, which may have provided additional insights or data on trauma-informed interventions. As a result, the findings may be influenced by publication bias and may not capture the full scope of existing work in this area. Second, this review was limited to interventions implemented within formal K-12 school settings and did not consider trauma-informed practices occurring in after-school programs or other informal educational environments. Third, the study is restricted to trauma-informed interventions implemented in the United States. This focus may limit the applicability of our findings to other global contexts. Future researchers are encouraged to examine interventions in other countries to broaden our understanding of how trauma-informed practices are adapted and implemented worldwide. Fourth, to be included in the present study, articles had to meet the criteria for a trauma-informed intervention as described by Avery and colleagues (2021). An analysis of the articles not meeting these criteria was outside of the scope of the present study, and future researchers could build on this review by systematically examining the literature to better understand misclassifications, gaps, and the broader landscape of what it means for an intervention to be trauma-informed.

Finally, to be included in this review, studies were required to examine at least one school violence outcome. This inclusion criterion may have excluded valuable research on trauma-informed interventions that focused solely on academic, behavioral, or mental health outcomes without specifically assessing their impact on school violence. Consequently, the findings may not reflect the full spectrum of effects that trauma-informed approaches can have within educational settings. Despite these limitations, the present study contributes to the growing body of literature by synthesizing available evidence on trauma-informed interventions and violence prevention in U.S. schools.

### Future Directions

There are several opportunities for future research to help contribute to the field and fill existing gaps in the literature on trauma-informed interventions and violence prevention in K-12 schools. First, there is a need for longitudinal studies to assess the long-term effects of trauma-informed programming on school violence outcomes, as most current research remains limited to cross-sectional or observational research designs. Given the lasting effect that trauma can have on youth development and overall well-being, further research is needed to understand the sustainability and long-term implications of trauma-informed interventions.

Second, this review revealed a paucity of studies focused on early childhood, with only one intervention that was implemented at the elementary level. Future efforts might consider prioritizing developmentally appropriate, trauma-informed approaches for elementary-aged students, including kindergarten. Additionally, future program evaluation must better address the needs of older adolescents in high school settings and expand trauma-informed supports across the multi-tiered systems of support framework, with a focus on targeted (Tier 2) and intensive (Tier 3) interventions for students displaying high-risk behaviors. Addressing trauma-informed approaches through a developmentally appropriate lens has the potential to better support students throughout their phases of development.

Third, researchers may consider moving beyond outcome evaluation to examine mediators and moderators of intervention effectiveness, including the use of path models to clarify how and for whom these strategies reduce school-based violence. An important, yet overlooked, component is the integration of cultural relevance; only 2 of the 7 studies in our sample described the importance of cultural relevance as a component of their trauma-informed intervention. Going forward, an intentional linkage between trauma-informed practices and cultural relevance is necessary to ensure interventions advocate for and affirm diverse school communities and student identities.

Finally, future researchers may consider addressing the inconsistent and variable results of trauma-informed interventions across different violence-related outcomes and diverse school contexts. For instance, while Dunn and colleagues (2020) reported improvements in students’ understanding of conflict resolution, there was no significant change in physical violence outcomes. Similarly, Swaim and colleagues (2008) found reductions in verbal victimization and violent intentions, but demonstrated no significant differences in physical victimization or verbal assaults. These findings underscore the complexity of school violence and the need for interventions across socioecological levels tailored to specific school contexts and student populations. The variability in program outcomes suggests that school climate, implementation, and cultural considerations play critical roles in determining outcomes.

## Conclusion

School-based trauma-informed practices demonstrate potential for reducing violence and fostering positive school climate, as evidenced by existing interventions that incorporate practice change, professional development, and organizational reform. However, there remains a critical need for rigorous research that systematically examines which components are most beneficial within a multi-tiered system of supports framework, from universal strategies that promote safety and trust throughout the school community, to targeted and intensive supports for students with higher risk or trauma exposure. By mapping current interventions and highlighting gaps in evidence, this scoping review points to the importance of integrating trauma-informed practices across all levels of support. Future studies may focus on evaluating outcomes, understanding mechanisms of impact, and identifying best practices for sustainable implementation.

## Supplementary Information

Below is the link to the electronic supplementary material.

Supplementary Material 1.
